# Dual-Message QR Codes

**DOI:** 10.3390/s24103055

**Published:** 2024-05-11

**Authors:** Kuo-Chien Chou, Ran-Zan Wang

**Affiliations:** Department of Computer Science & Engineering, Yuan Ze University, 135 Far-East Rd., Chung-Li, Taoyuan 320, Taiwan; s1099102@mail.yzu.edu.tw

**Keywords:** QR code, nested QR code, two-layer QR code, dual-message QR code, 2D barcode

## Abstract

A novel dual-message QR code is proposed for carrying two individual messages that can be read by standard QR code readers: one from a close range and the other from a large distance. By exploring the module value determining the rule of typical QR code readers, we designed two-state module blocks that can be recognized as different module values through changing the distance from which the QR code is scanned, and applied them to construct the proposed dual-message QR code. Experiments were conducted to test the readability of the two messages within a dual-message QR code, with the results demonstrating the high feasibility of the proposed method. The dual-message QR code can be applied for designing creative applications. For example, an interactive wedding card that can access the growing film of the groom and that of the bride interchangeably, which bring the viewers a higher-quality experience.

## 1. Introduction

The quick response code (QR code) [1,2] is the most popular and widely used 2D barcode in the world. It is a kind of contactless data acquisition method with several favorable properties, including high-capacity data storage, high tolerance against partial corruption, readability from any orientation, and rapid decoding. A QR code provides a publicly available and user-friendly interface for facilitating automatic identification and data capture, and can be applied to identify, locate, and track items in diverse fields such as manufacturing, transportation, healthcare, office automation, retail, and advertising.

Most contemporary smartphones can read QR codes using their built-in cameras combined with publicly available QR applications (apps) downloaded from the Internet. In a typical QR code app, the camera app of the phone is launched to capture an image encompassing a QR code, the region of the QR code is then identified and extracted, and the message in the QR code is decoded. A predefined action is usually performed according to the decoded message, such as a URL message launching a browser on the phone to access a target website, or an SMS (Short Message Service) message being sent to the phone. This functionality provides a rapid method for accessing the target app without cumbersome typing, and is an effective and popular interface for commercial advertising and publicity strategies.

In order for QR code apps to exhibit robustness, some research [3,4,5] has applied image processing algorithms to increase the decoding rate of QR code images with non-uniform backgrounds or viewed in variable lighting conditions. A standard QR code consists of a square grid of black and white square blocks that reveals no useful information to human eyes. Some QR code generation methods [6,7,8,9,10,11,12] were proposed for constructing QR codes with visually meaningful patterns in order to provide discriminative information to human beings.

A standard QR code encodes a canonical public text string that can be read by standard QR code apps. To deliver sensitive data to intended users via QR codes, some methods for hiding data [13,14,15,16,17] have been proposed for concealing additional private data in QR codes. Some other methods [18,19,20] have been reported for increasing the amount of data in a QR code. However, specific QR code readers with dedicated decoding algorithms are required to access the additional data included by applying these methods.

In 2019, Yuan et al. [21] proposed a two-layer QR code that encodes two messages in a fabricated QR code comprising an upper translucent layer and a lower opaque layer. The two messages can be accessed by taking two photographs of the QR code: one from the left and the other from the right. However, a physical fabrication process is required to construct a two-layer QR code, which increases the cost and time. We have previously proposed the nested QR code [22] that comprises outer and inner QR codes, with these two codes being read separately by slightly adjusting the distance and/or angle when capturing images. In this method, the inner QR code is constrained to a small region of the outer QR code, and this approach has the disadvantage of decreasing the robustness of the decoding process against partial corruption. 

Encoding two individual messages on a single QR code provides a friendly interface for delivering two individual but closely related messages. For example, given a QR code that encodes two URLs, one links to the growing film of the groom and another links to that of the bride. By changing the distance to scan, the QR code on the wedding card can access the growing film of the groom and that of the bride interchangeably, which will create a quality experience for the guests attending the wedding party. Given another example, a company can design a QR code that encodes two URLs, providing two types of media related to the company: one links to the website of the company, and the other links to an introductory video of the company on YouTube.

This paper presents a new method for integrating two individual QR codes within a square region. The two QR codes can be read by standard QR code readers: one from a close range and the other from a large distance. The two QR codes are the same size and can be displayed or printed without requiring a specific fabrication process. This approach provides an economical and robust way to develop practical applications that deliver two messages via a single QR code that can easily be read using popular QR code apps in the marketplace.

The remainder of this paper is organized as follows: Section 2 introduces the main features and decoding process of standard QR codes, Section 3 describes the proposed method, Section 4 presents experimental results, and conclusions are drawn in Section 5.

## 2. QR Code Features and Decoding Process

A standard QR code stores a string of text that can be read by any typical QR code scanning program. In this section, the features of a standard QR code are first described, and then the decoding process of a typical QR code scanner is introduced. These introductions provide readers with the background knowledge that makes the subsequent research methods easier to grasp.

### 2.1. QR Code Features

A QR code is a square image comprising a grid of black (or dark) and white (or light) square blocks called modules. Each module represents 1 bit of data, with a white module representing a 0 and a black module representing a 1. There are 40 versions of QR codes, and a QR code of version *v* (ranging from 1 to 40) has (17 + 4*v*) × (17 + 4*v*) modules. Four standardized encoding modes (numeric, alphanumeric, byte, and kanji) are specified in the QR code standard for encoding text consisting of different character sets. The text is encoded using Reed–Solomon codes to provide an error-correction capability. Four error-correction levels (L, M, Q, and H) are specified, which have error-correction capabilities of about 7%, 15%, 25%, and 30%, respectively [1].

**Figure 1:**
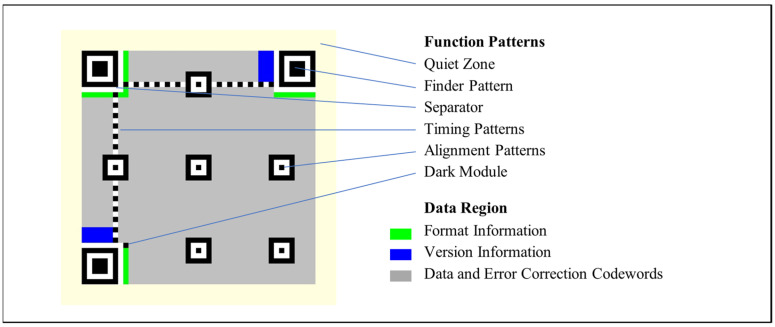
Structure of a version-7 QR code. Function patterns are used to locate and identify the parameters of a QR code. Data modules contain the input-data code words and the error-correction code words.

Figure 1 shows the structure of a standard QR code of version 7. The modules of a QR code can be classified into function patterns and the data region. Function patterns include finder patterns, alignment patterns, timing patterns, separators, a dark module, and the quiet zone, which are designed to help identify and locate a QR code and to perform module alignment. The data region contains the actual data and the error-correction code words encoded in the QR code.

### 2.2. QR Code Decoding Process

**Figure 2:**
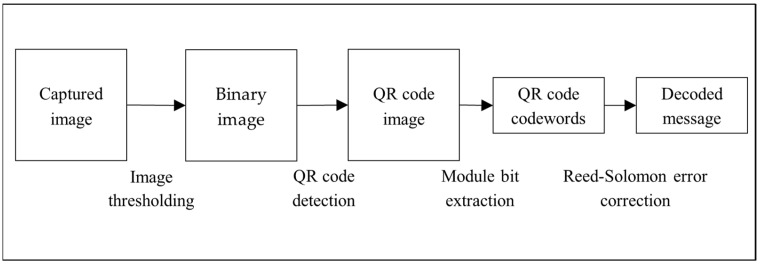
Typical QR code decoding procedure.

The typical QR code decoding process is illustrated in Figure 2, which consists of the following four steps: (1) image thresholding, (2) QR code detection, (3) module bit extraction, and (4) error correction.

The QR code standard defines black and white modules, but it does not specify the image thresholding criterion for determining whether a specific module block is a black module or a white module. According to previous reports [4,10], local thresholding methods are more robust against illumination variations than global thresholding methods, and so they are often adopted for thresholding the captured image in QR code apps.

The finder patterns located at the three corners of a QR code are key structures for detecting the presence of the code. After the finder patterns have been detected, the estate of the QR code is located and normalized into a standard square by applying a perspective transformation to correct the distortion introduced by capturing images from different angles and distances. A module bit extraction process is performed to determine the module values of all module blocks. The extracted module values in the data region constitute the input-data and error-correction code words of the QR code.

The data extracted from module blocks might contain errors due to the image being distorted or performing inappropriate thresholding, and so Reed–Solomon error correction is applied until a valid text string is obtained.

## 3. Proposed Method

The QR code standard defines a module to be a square image representing bit data. Typically, a black module represents a bit value of 1 and a white module represents a bit value of 0. However, the criterion for determining a square image as a black module or a white module is not specified in the QR code standard. A model to characterize the rule for determining the module value for general QR code readers is next established below.

Given a module block B, the module value of B can be determined by evaluating the average luminance of a centroid region Ω of B:

(1) $$module\_value(B)=\left\{\begin{array}{c} 0\;if\;\lambda_{\Omega }(B)\geq t,\\ 1\;if\;\lambda_{\Omega }(B)<t, \end{array}\right.$$

where *t* is the threshold and $\lambda_{\Omega }(B)$ is the average luminance of pixels {*p_ij_*} in centroid region Ω of *B* calculated as

(2) $$\lambda_{\Omega }(B)=\frac{1}{number\;of\;pixels\;in\;\Omega }\sum_{p_{ij\in \Omega }}{\lambda (p}_{ij}).$$

Most aesthetic QR codes [8,9,10,11,12] in the literature were designed on the basis that the module value is determined by a one-third centroid region (i.e., Ω = 1/3) of a module block. Experimental results demonstrated that the generated QR codes performed well both in terms of the quality of the visual elements and decoding robustness of the QR codes. However, we observe that most functional QR code readers do not evaluate the module value based on the average luminance of all pixels in a one-third centroid region of a module block. 

**Figure 3:**
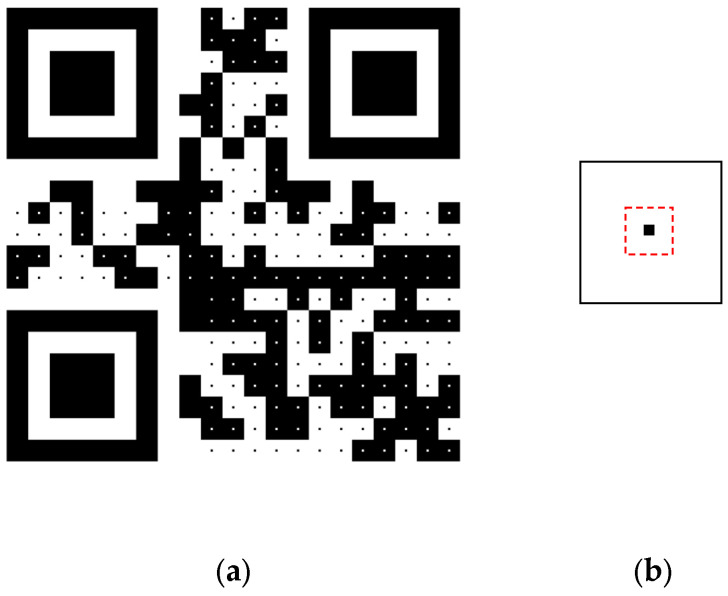
Special QR code for exploring the module value determination rule. (**a**) The testing QR code. (**b**) Layout of a module block.

To explore the module value determination rule adopted in functional QR code readers in smartphones, a special QR code was created by drawing all modules in the data region in such a way that only those pixels within a small centroid region were set to the module color, and other pixels were set to the inverse module color (white changed to black, and black changed to white), as shown in Figure 3.

Figure 3a shows a test QR code encoding the text “QR Code” that was generated using a module size of 29 × 29 pixels and a centroid size of 3 × 3 pixels. The structure of a module block is shown in Figure 3b, where the one-third centroid region of a module is marked using a dotted square. It can easily be calculated that the centroid region of a module occupies only about 10% of the one-third centroid region of a module block. If the module value is evaluated using the average luminance of all pixels in the one-third centroid region of a module, all of the module values in the data region will be invalid, and the message cannot be decoded correctly using QR code readers. To test this, this page of the paper was (1) displayed on a computer screen and (2) printed on an A4 sheet of paper, and we scanned the QR code using various QR code readers, including the iPhone X and iPhone 13 camera and the top-five most downloaded QR Code apps in both the Apple Store and Android Market. Experiments showed that all of the tested QR code readers could decode the “QR Code” message correctly, although sometimes several trials moving the camera around the QR code were needed. 

**Figure 4:**
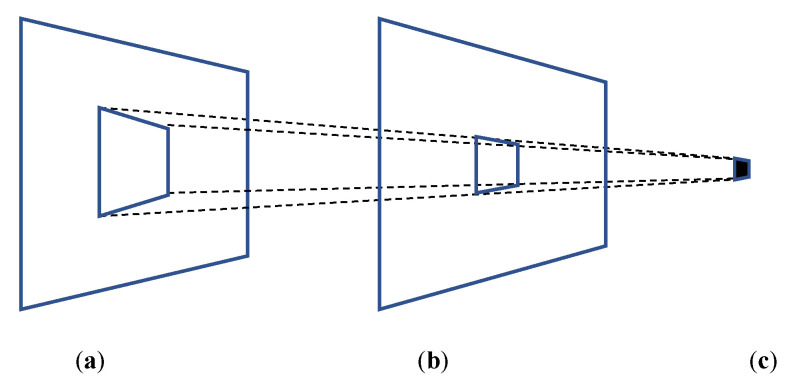
Illustration of pixels of an exhibited module block that are projected onto the centroid pixel of a module block in the captured image. (**a**) QR code module located at a distance. (**b**) QR code module located at close range. (**c**) A pixel in the camera image plane.

A model of the module value determination rule was established based on the ZXing [23] barcode scanning library, which contains QR code encoding/decoding functions and is widely used in both academic research and commercial applications. We examined the source code of the QR code decoding program in that library, and observed that only the centroid pixel of a module block in the captured image is utilized to determine the module value. Based on the image projection principle, the centroid pixel of a module block in the captured image is projected from a region of pixels of the corresponding module block onto the exhibited QR code, as illustrated in Figure 4. This figure shows that the pixels projected onto the centroid pixel of a module block vary with the scanning distance: if the QR code is scanned from close range, as shown in Figure 4b, a small region of pixels is projected onto the centroid pixel, whereas if the QR code is scanned from a large distance, a larger region of pixels is projected onto the centroid pixel. 

The following QR code module value determination rule is established based on the previous observation:

**Property 1.**  *The centroid region of pixels of a module block in the exhibited QR code is applied to evaluate the module value. However, the region is not constant: pixels within a small centroid region are used in determining the module value if the QR code is scanned from close range, while pixels in a large centroid region are utilized in determining the module value if the QR code is scanned from a large distance.*

The lack of a standard specification for the module value evaluation criteria in the QR code decoding process means that different QR code apps may apply different extraction methods. Moreover, the decoding algorithms used in most commercial apps are not publicly disclosed. In practical implementations of QR code readers such as the ZXing [23] barcode scanning library, the centroid pixel of each QR code module block in the captured image is used to determine the module value, which provides rapid decoding and good robustness. Based on the perspective property inherent in image projection previously discussed above, an object that is further from the camera will tend to appear smaller in the image plane. In other words, a pixel in the image plane is projected from a small region of the scene at a close range, and from a larger region of the scene at a distance. In short, if the QR code is scanned from a close range, a small region of pixels within the exhibited module block is sampled to determine the module value; however, if the QR code is scanned from a large distance, a larger region of pixels within the exhibited module block is sampled to determine the module value. 

The properties of the method used to determine the module value as discussed above enable the design of a module block that can be recognized as different module values by QR code readers. This is referred to as a two-state module block, as defined in the following:

**Definition 1.**  *A near–far, two-state module block is a module block that can be decoded as a near = {0, 1} module by QR code readers when the QR code is scanned from a close range, and can be decoded as a far = {0, 1} module by QR code readers when the QR code is scanned from a large distance.*

The fundamental idea underlying the dual-message QR code is to set the color of a small centroid region ω × ω of a module block to the module value to be decoded at a close range, and the color of the other outer region of the module block to the module value to be decoded at a distance. Table 1 summarizes the structure of four types of two-state module blocks according to the combination of module values to be decoded at a close range and at a distance; for example, in a 0-1 two-state module block, the small centroid region is set to white and the outer region is set to black.

**Figure 5:**
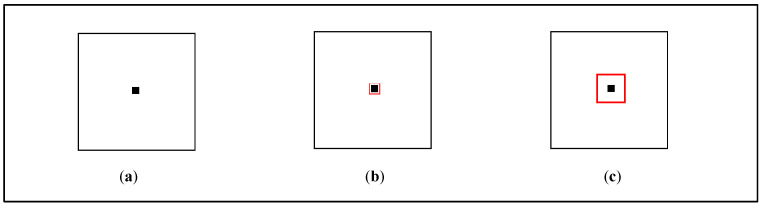
Working model of the two-state module block. (**a**) The designed 1-0 two-state module block. (**b**) The region to be projected onto the centroid pixel of the module block from a close range. (**c**) The region to be projected on the centroid pixel of the module block from a large distance.

Figure 5 illustrates the working model of the designed two-state module block. Figure 5a is a 1-0 two-state module block. The region of pixels in the exhibited module block to be projected onto the centroid pixel of the module block in the image plane from a close range and from a large distance are marked by the red square in Figure 5b and Figure 5c, respectively. It can easily be evaluated that the average luminance within the red centroid region in Figure 5b is lower than the threshold, while that in Figure 5c is higher than the threshold. Therefore, the module block will be recognized as a 0 module from a close range and as a 1 module from a large distance.

Based on the two-state module blocks, the proposed dual-message QR code is summarized next:
**Dual-Message, QR-Code-Generating Algorithm****Input:**   MSG_near_: text string to be read from a close range.   MSG_far_: text string to be read from a large distance. *m*: side length of a module block specified by the user; the size of a module block is *m* × *m* pixels.   ω: side length of the centroid region specified by the user; the size of the centroid region is ω × ω pixels.**Output:**   QR_dual_: the dual-message QR code.**Steps:**   Step 1. Encode MSG_near_ in the binary representation of QR code QR_near_, and encode MSG_far_ in the binary representation of QR code MSG_far_. QR_near_ and QR_far_ are standard QR codes with the same version and error-correction level.   Step 2. Fetch a not-processed-yet module bit *p* in the scanning order from QR_near_ and corresponding module bit *q* from QR_far_, and draw the module on QR_dual_ using a *p*-*q* two-state module block.   Step 3. Repeat Step 2 until all modules in QR_near_ and QR_far_ are processed.   Step 4. Add a quiet zone border with a width of 4*m* to QR_dual_.End.

## 4. Experimental Results

**Figure 6:**
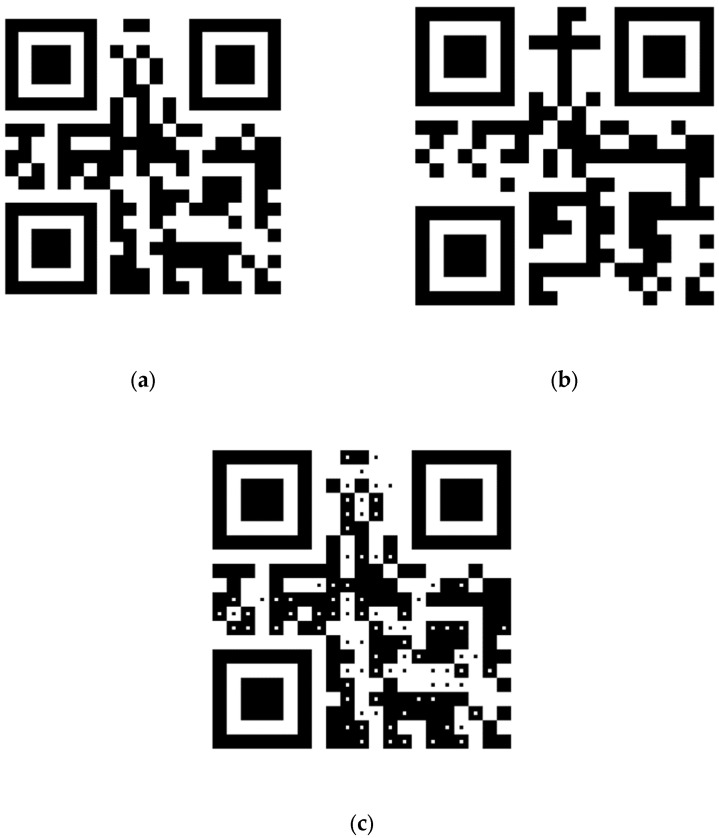
An example of dual-message QR code. (**a**) Standard QR code with message “Near view is less.” (**b**) Standard QR code with message “Far view is more.” (**c**) Dual-message QR code generated by setting the module block size and the centroid region size to 29 × 29 and 7 × 7 pixels, respectively.

Figure 6 shows an example of dual-message QR code encoding two separate messages using the proposed method. The QR code in Figure 6a is a typical QR code with message MSG_near_ = “Near view is less,” and The QR code in Figure 6b is a typical QR code with message MSG_far_ = “Far view is more.” The two QR codes are both version-1 standard QR codes with error-correction level set to L. The QR code in Figure 6c is the dual-message QR code that encodes both messages MSG_near_ and MSG_far_. Parameters *m* and ω applied to generate the dual-message QR code are set to 29 and 7, respectively.

**Figure 7:**
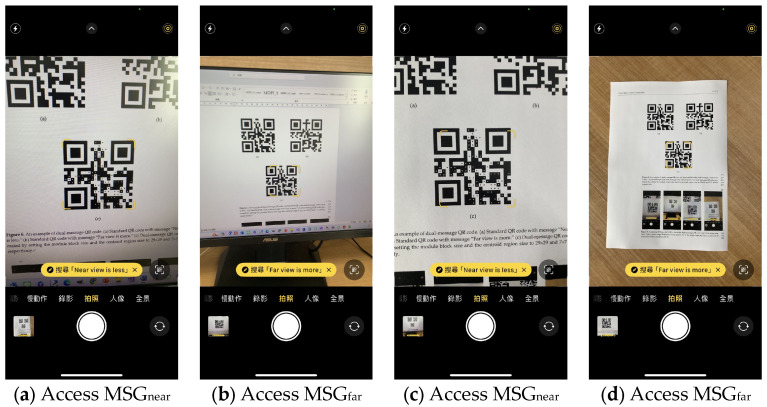
Accessing MSG_near_ and MSG_far_ from the dual-message QR code. (**a**) Snapshot of QR_dual_ displayed on the screen captured from a close range. (**b**) Snapshot of QR_dual_ displayed on the screen captured at a distance. (**c**) Snapshot of QR_dual_ displayed on paper captured from a close range. (**d**) Snapshot of QR_dual_ displayed on paper captured at a distance.

The dual-message QR code was (1) displayed on the screen of an ASUS VX239H Monitor with resolution 1920 × 1080 pixels, and (2) printed out on an A4 paper so that the QR code had a size of 4.0 × 4.0 cm^2^. Figure 7 shows four snapshots of the QR code detected in the build-in camera of an iPhone X smartphone. Figure 7a is the snapshot to QR_dual_ displayed on the screen captured from a close range about 30 cm to the screen, on which we can see the message MSG_near_ = “Near view is less” was read by the QR code reader on the built-in camera. Figure 7b is the snapshot to QR_dual_ displayed on the screen captured at a distance about 50 cm apart from the screen, on which the message MSG_far_ = “Far view is more” was decoded. Figure 7c is the snapshot to QR_dual_ displayed on paper captured from a close range about 20 cm to the paper, on which the message “Near view is less” was read. Figure 7d is the snapshot to QR_dual_ displayed on paper captured at a distance about 40 cm apart from the paper, on which the message “Far view is more” was decoded. We also tested the reading of message from QR_dual_ using iPhone 13 built-in camera and popular QR code readers downloaded from Android Market installed on a Samsung A53 Android smartphone, and the results show MSG_near_ and MSG_far_ can easily be read separately from a close range and at a large distance using all of QR code scanners. The readers can do this test using any standard QR code reader in his/her smartphone. Note that the built-in camera of Samsung A53 Android smartphone does not work well in decoding MSG_near_ due to its specific QR code decoding algorithm. In our test, it consistently tends to read MSG_far_ instead.

**Figure 8:**
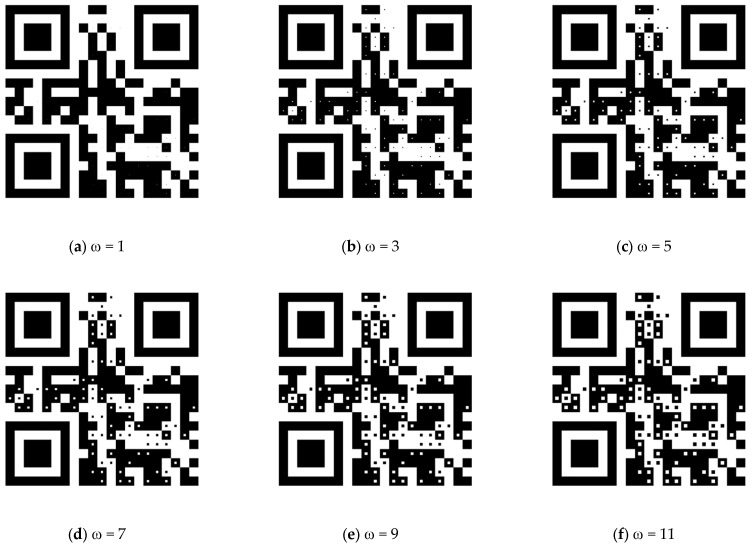
Dual-message QR codes generated with module block size of 29 × 29 and vary centroid region size from 1 to 11.

To select an appropriate ω that allows both MSG_near_ and MSG_far_ to be easily decoded by standard QR code readers, two-state module blocks with centroid regions of different sizes were applied to construct the dual-message QR code in Figure 8. The readability of MSG_near_ and MSG_far_ was tested using four popular QR code readers: (1) the build-in camera of an iPhone X, (2) the Barcode Scanner app on a Samsung A53 Android smartphone, and the QR Scanner app on both (3) an iPhone X and (4) a Samsung A53 Android smartphone. In this experiment, the QR codes were printed on paper so that they each had a size of 6.0 cm × 6.0 cm. Six users were asked to scan MSG_near_ and MSG_far_ from the dual-message QR code using the four QR code readers, and a message was defined as decodable by the QR code reader if all of the users were able to successful read it using the QR code reader within 8 s, and undecodable otherwise. 

**Figure 9:**
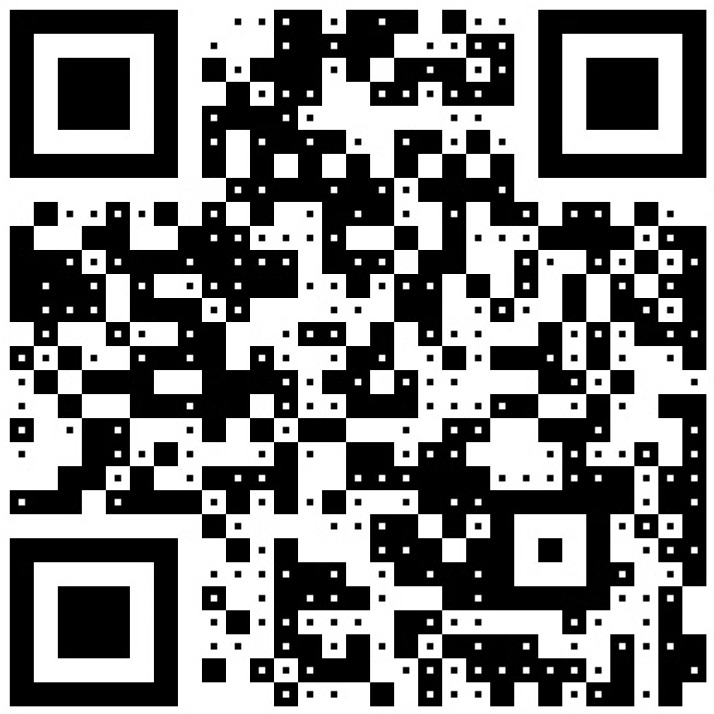
Another example of dual-message QR code with two messages “https://youtu.be/MUcTeoFJ9qo” and “https://youtu.be/zNmhCabrJlg.” The module block size and the centroid region size are set to 11 × 11 and 3 × 3 pixels, respectively.

Figure 9 is another example of dual-message QR code encoding MSGnear = “https://youtu.be/MUcTeoFJ9qo” and MSGfar = “https://youtu.be/zNmhCabrJlg.” The two messages are two URLs linking to the growing up films of a new couple: one for the bride and another for the groom. Both of them are encoded using version-3 QR code with an error-correction level of L. Parameters *m* and ω applied to generate the dual-message QR code are set to 11 and 3, respectively. 

Table 2 summarizes the results of this experiment. It can be seen that both MSG_near_ and MSG_far_ were decodable in the cases where the centroid region size was set to 5 × 5, 7 × 7, and 9 × 9 pixels. The QR code readers tended to decode MSG_near_ from the dual-message QR code if a larger ω was set, while they tended to decode MSG_far_ if a smaller ω was set. In our experience, a size of slightly smaller than one-third of the side length of a module block is the best choice of ω for constructing a dual-message QR code.

Table 3 compares the properties of the proposed dual-message QR code with QR code schemes carrying two levels of messages that can be accessed by standard QR code readers. A previously reported method [21] accesses the two messages by viewing from the left and from the right. The two QR codes are of the same size, but it is difficult to access the messages when they can only be viewed from narrow range of directions, and a physical fabrication process is required to generate the two-layer QR code. The nested QR code [22] can be generated with less effort by printing it out directly, and the two messages can be accessed by slightly adjusting the distance and angle to capture the corresponding QR code image. However, the inner QR code is constrained to a small region of the outer QR code. In contrast, the proposed dual-message QR code encodes two messages in equal-size QR codes, which can be read by simply changing the distance at which the image is captured: one from a close range and the other from a large distance. Moreover, generating a dual-message QR code is simple and rapid, and the two messages that it contains can be accessed with good robustness.

## 5. Conclusions

This paper presents a new method for encoding two individual messages in a single QR code, where the two messages can be read using standard QR code apps: one from a close range and the other from a large distance. Construction methods for the dual-message QR code are provided, and the code can be printed out or displayed without a fabrication process. The proposed dual-message QR code extends convention QR codes that deliver a single message to two tracks of messages, which has applications in many creative fields.

## Figures and Tables

**Table 1:** Structure of near–far, two-state module blocks.

View Distance	Value	Value	Value	Value
Near	0	0	1	1
Far	0	1	0	1
Module block	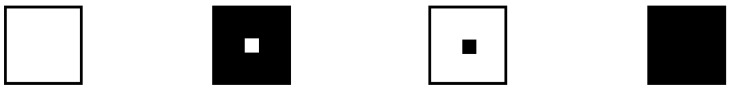			

**Table 2:** Decoding to MSG_near_ or MSG_far_ from dual-message QR codes with different centroid sizes of a 29 × 29 module block.

ω × ω	1 × 1	5 × 5	3 × 3	7 × 7	9 × 9	11 × 11
iPhone camera	x/x	o/o	o/o	o/o	o/o	o/x
iPhone QR Scanner	x/x	o/o	o/o	o/o	o/o	o/o
Android Barcode Scanner	x/x	x/o	o/o	o/o	o/o	o/x
Android QR Scanner	x/x	x/o	o/o	o/o	o/o	o/x

o: decodable; x: undecodable.

**Table 3:** Comparison of QR code methods with two-layer data that can be accessed using standard QR code readers.

	Layer-1 Message	Layer-2 Message	Sizes of Two QR Codes	Generation Cost of QR Codes
[21]	Left view	Right view	Equal	High
[22]	Inner	Outer	Different	Low
Ours	Close range	From a large distance	Equal	Low

## Data Availability

Data are contained within the article.
